# CRISPR Screen Identifies the RNA-Binding Protein Eef1a1 as a Key Regulator of Myogenesis

**DOI:** 10.3390/ijms25094816

**Published:** 2024-04-28

**Authors:** Weiwei Liu, Wei Wang, Zishuai Wang, Xinhao Fan, Wangchang Li, Yuxin Huang, Xiaogan Yang, Zhonglin Tang

**Affiliations:** 1 Guangxi Key Laboratory of Animal Breeding, Disease Control and Prevention, College of Animal Science & Technology, Guangxi University, Nanning 530004, China; liuweiwei@st.gxu.edu.cn (W.L.); liwangchang@st.gxu.edu.cn (W.L.); 2118391010@st.gxu.edu.cn (Y.H.); 2 Kunpeng Institute of Modern Agriculture at Foshan, Agricultural Genomics Institute at Shenzhen, Chinese Academy of Agricultural Sciences, Foshan 528226, China; wwlucky1005@webmail.hzau.edu.cn (W.W.); wangzishuai@caas.cn (Z.W.); fanxinhao@caas.cn (X.F.); 3 Key Laboratory of Agricultural Animal Genetics, Breeding and Reproduction of Ministry of Education & Key Lab of Swine Genetics and Breeding of Ministry of Agriculture and Rural Affairs, Huazhong Agricultural University, Wuhan 430070, China; 4 Shenzhen Branch, Guangdong Laboratory for Lingnan Modern Agriculture, Key Laboratory of Livestock and Poultry Multi-Omics of MARA, Agricultural Genomics Institute at Shenzhen, Chinese Academy of Agricultural Sciences, Shenzhen 518124, China

**Keywords:** CRISPR screen, Eef1a1, RNA-binding protein, myogenesis

## Abstract

Skeletal muscle myogenesis hinges on gene regulation, meticulously orchestrated by molecular mechanisms. While the roles of transcription factors and non-coding RNAs in myogenesis are widely known, the contribution of RNA-binding proteins (RBPs) has remained unclear until now. Therefore, to investigate the functions of post-transcriptional regulators in myogenesis and uncover new functional RBPs regulating myogenesis, we employed CRISPR high-throughput RBP-KO (RBP-wide knockout) library screening. Through this approach, we successfully identified Eef1a1 as a novel regulatory factor in myogenesis. Using CRISPR knockout (CRISPRko) and CRISPR interference (CRISPRi) technologies, we successfully established cellular models for both CRISPRko and CRISPRi. Our findings demonstrated that Eef1a1 plays a crucial role in promoting proliferation in C2C12 myoblasts. Through siRNA inhibition and overexpression methods, we further elucidated the involvement of Eef1a1 in promoting proliferation and suppressing differentiation processes. RIP (RNA immunoprecipitation), miRNA pull-down, and Dual-luciferase reporter assays confirmed that miR-133a-3p targets Eef1a1. Co-transfection experiments indicated that miR-133a-3p can rescue the effect of Eef1a1 on C2C12 myoblasts. In summary, our study utilized CRISPR library high-throughput screening to unveil a novel RBP, Eef1a1, involved in regulating myogenesis. Eef1a1 promotes the proliferation of myoblasts while inhibiting the differentiation process. Additionally, it acts as an antagonist to miR-133a-3p, thus modulating the process of myogenesis.

## 1. Introduction

Skeletal muscle, a dynamic tissue composed of multinucleated myofibers, comprising approximately 30% to 40% of their total body weight, plays a pivotal role in various physiological processes, including locomotion, posture maintenance, and metabolic homeostasis [1,2]. Despite their crucial role in maintaining health and vitality, skeletal muscles are susceptible to various disorders [3]. The intricate development of skeletal muscle, known as myogenesis, is a complex phenomenon encompassing the sequential activation and repression of numerous genes, tightly regulated by intricate molecular mechanisms [4,5,6]. This process involves the orchestrated progression of skeletal muscle precursor cells’ proliferation, differentiation, and fusion, resulting in the formation of mature, multinucleated muscle fibers [7,8]. Paired box 3 (Pax3), paired box 7 (Pax7) [9,10], and myogenesis regulator factors (MRFs) form a key regulatory network during skeletal muscle development [11,12]. Additionally, the family of myogenic regulatory factors includes muscle-specific regulatory factor 4 (MRF4), myogenic factor 5 (Myf5), myoblast determination factor (MyoD), and myogenin (MyoG). However, our focus lies in post-transcriptional regulatory mechanisms. The post-transcriptional regulation of gene expression is intricately governed by an ensemble of factors, including non-coding RNAs like miRNAs, circRNAs, and lncRNAs [13]. These molecules exert their influence by modulating RNA stability, translation, and splicing, thereby contributing to the complex orchestration of biological processes in myogenesis [14,15,16,17,18]. Among this diverse landscape of post-transcriptional regulators, our particular focus centers on a distinct category: RNA-binding proteins (RBPs). Based on our understanding, investigations into the role of RBPs in the context of skeletal muscle development have been relatively scarce [19]. RBPs bind to specific RNA sequences and structures, impacting mRNA stability, translation, and localization, thus regulating gene expression and influencing cell fate decisions [20,21,22]. The regulatory mechanisms mediated by RBPs are particularly crucial in determining cell lineage commitment and tissue-specific differentiation. These processes are of fundamental importance for understanding muscle development and hold great promise for therapeutic interventions in muscle-related disorders.

The CRISPR-associated nuclease Cas9 genome editing system has demonstrated remarkable effectiveness in conducting high-throughput loss-of-function screens. Numerous studies have adeptly utilized this tool to explore genetic factors associated with drug and toxin resistance [23], cancer metastasis [24,25], and immune responses [26,27]. In contrast to RNA interference (RNAi)-based gene knockdown approaches, the CRISPR/Cas9-based gene knockout method offers the more comprehensive suppression of target gene expression, with reduced off-target effects, especially when employing carefully designed guide RNAs [28]. Moreover, the potency of CRISPRi and CRISPRa high-throughput library screening modes have been seamlessly incorporated into this framework [29,30,31]. This integration further enhances the versatility of CRISPR-based screening approaches, enabling nuanced control over gene expression and expanding the horizons of functional analysis in numerous research domains.

While the functions of transcription factors and non-coding RNAs in myogenesis have been extensively documented, the role of RNA-binding proteins (RBPs) in this process has hitherto remained ambiguous. Through utilizing CRISPR high-throughput RBP-KO (RBP-wide knockout) library screening, our study aims to screen and validate potential RNA-binding proteins that regulate skeletal muscle myogenesis, providing new candidate genes for muscle development. It also aims to decipher the specific function of the RNA-binding protein Eef1a1 and understand its regulatory mechanisms in myogenesis. Here, we first constructed an RBP knockout cell library and utilized CRISPR high-throughput screening to identify, for the first time, the proliferation phenotype of myoblasts. Additionally, we investigated new functions and regulatory mechanisms of Eef1a1 in regulating myogenesis.

## 2. Results

### 2.1. Identifying RNA-Binding Protein Eef1a1 as a Novel Regulator for Myogenesis

**Figure 1:**
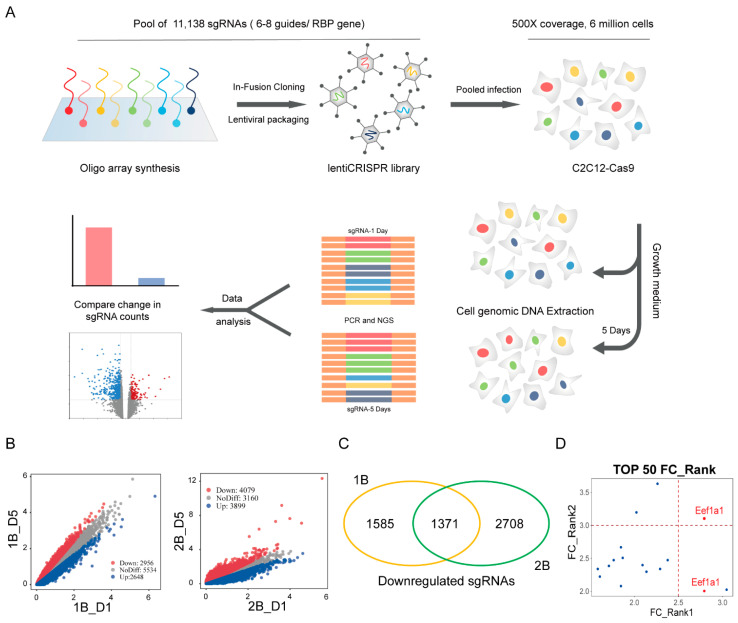
CRISPR screen reveals involvement of Eef1a1 in cellular proliferation. (**A**) Schematic representation depicting the mode of CRISPR high-throughput library screening for identifying key RNA-binding proteins (RBPs) in C2C12. This library contains 11,138 sgRNAs targeting 1542 genes, each with 6–8 sgRNAs per gene. (**B**) Dual volcano plots illustrating differential sgRNA abundance in biological replicates 1B (flow cytometry) and 2B (drug selection). D1 represents the control sample on day 1 and D5 represents the experimental sample on day 5. The volcano plots depict variations in sgRNA quantities, highlighting distinct patterns between the two experimental conditions. (**C**) Venn diagram showcasing the overlap of significantly downregulated differential sgRNAs enriched in two independent biological replicates. A total of 1371 common sgRNAs exhibit substantial downregulation across both experimental sequences. (**D**) Quadrant plot displaying the distribution of 16 significantly differentially expressed sgRNAs.

To systematically identify novel regulators of myogenesis and investigate functional RNA-binding proteins (RBPs) involved in skeletal muscle development, we conducted a loss-of-function cell library targeting RNA-binding proteins. We screened for genes essential for the proliferation of C2C12 myoblasts, a mouse muscle cell line, as illustrated in Figure 1A.

First of all, we generated a Cas9-expression C2C12 cell line using a Cas9-expressing lentiviral plasmid. And also, we constructed a lentiviral RBP single guide RNA (sgRNA) library for packaging lentivirus. This library contains 11,138 sgRNAs targeting 1542 genes, with each gene having 6–8 sgRNAs. Additionally, 120 sgRNAs were included as controls, and the sgRNA sequences can be found in the Table S1. The list of 1542 genes targeted by the 12K sgRNA library synthesized using CustomArray arrays has been provided in Tables S2 and S3. And then, we infected Cas9-expression C2C12 cells with the lentiviral RBP sgRNA library for CRISPR gene editing. The lentivirus infection was performed at a multiplicity of infection in order to retain a 500-fold representation of the library. After obtaining a sufficient number of positive cells through flow cytometry (1B) and drug selection (2B), each biological replicate was divided into two groups. Half of the positive cells were harvested as the day 1 control sample, while the remaining cells were cultured continuously in growth medium for an additional 5 days to serve as the day 5 sample. The premise of our CRISPR screening was that when a gene was knocked out in a cell, the cell proliferated faster, resulting in the enrichment of the cell population. In this way, we expected to screen out candidate genes related to skeletal muscle cell proliferation.

The differential analysis of sgRNAs revealed that 2956 sgRNAs were significantly down-regulated with the drug selection method and 4079 with the FACS-based approach (Figure 1B). The full list of differentially enriched sgRNAs for Venn D1 vs. D5 has been provided in Table S4. Notably, a Venn diagram analysis combined both strategies, identifying 1371 sgRNAs significantly down-regulated in both datasets (Figure 1C). From this pool of 1371 sgRNAs, we selected the top 50 differentially ranked sgRNAs from each biological replicate. An intersection analysis unveiled a promising group of 16 candidate sgRNAs, including two targeting the Eef1a1 gene. These specific sgRNAs demonstrated statistically significant down-regulation (Figure 1D). These findings motivated us to focus our research on Eef1a1 as a candidate gene. We were dedicated to validating and exploring its crucial role in the complex myogenesis process.

### 2.2. Conserved Eef1a1 Regulates Myogenesis and Muscle Regeneration

In order to unravel the intricacies of Eef1a1 involvement in myogenesis, we embarked on a comprehensive characterization of this pivotal protein. Leveraging data from the National Center for Biotechnology Information (NCBI) database, we determined that the amino acid sequence within the coding sequence (CDS) region of Eef1a1 was highly conserved across human, mouse, and pig species. Among the 462 amino acids scrutinized (Figure S1), only a singular amino acid (Serine) deviated from the other two species, which featured Asparagine at that specific position. This observation underscored the remarkable conservation of the Eef1a1 sequence across these species.

**Figure 2:**
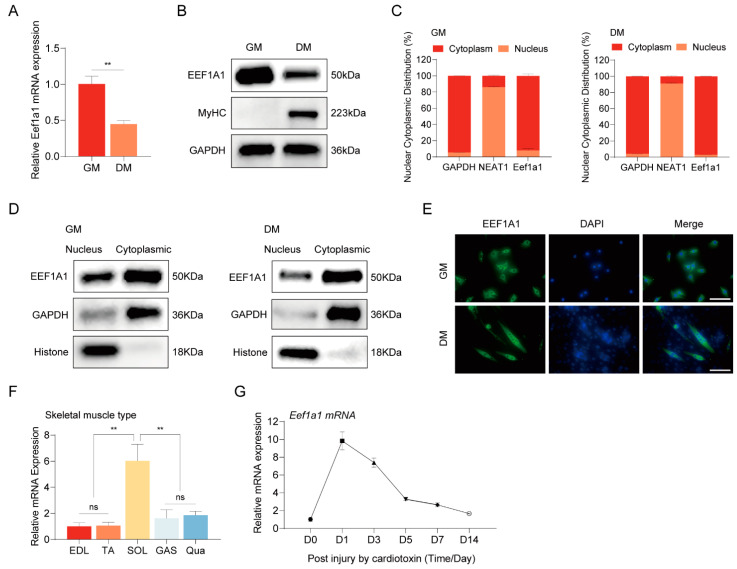
Characterization of Eef1a1 in myogenesis and muscle regeneration. (**A**) RT-qPCR and (**B**) Western blot analyses illustrating the down-regulation of Eef1a1 expression during myogenic differentiation. (**C**) RT-qPCR and (**D**) Western blot investigations revealing the cytoplasmic and nuclear distribution of Eef1a1 in proliferating C2C12 myoblast cells (GM) and cells differentiated for 3 days (DM) in differentiation medium. (**E**) Immunofluorescence assay demonstrating the subcellular localization of Eef1a1 in cytoplasm and nuclei. Magnification 40×, scale bar 100 µm. (**F**) The expression levels of Eef1a1 in different fiber types in mice. EDL (extensor digitorum longus), TA (tibialis anterior), SOL (soleus), GAS (gastrocnemius), and Qua (quadriceps) muscles. Each group comprises 3–5 biological replicates of mice. (**G**) Expression patterns of Eef1a1 in TA muscle of mice during the process of muscle regeneration. The RT-qPCR results were normalized against GAPDH expression. Error bars represent standard deviation and were derived from three independent experiments. ** *p* < 0.01; ns, not significant.

Our investigation then turned to the dynamic expression of Eef1a1 during distinct phases of cellular development. Employing RT-qPCR and Western blot techniques, we assessed Eef1a1 expression in C2C12 myoblasts, both during proliferation (day 0 [D0]) and differentiation (day 3 [D3]). Our findings revealed a higher expression of Eef1a1 during the proliferation phase compared to the differentiation phase (Figure 2A,B). This variation in expression implies a potential shift of Eef1a1’s functional significance as cells traverse diverse stages of proliferation and differentiation.

To gain deeper insights into the subcellular localization of Eef1a1, we conducted nuclear and cytoplasmic fractionation assays. Our analyses consistently indicated that Eef1a1 predominantly localizes to the cytoplasm, as evidenced by both RNA and protein levels (Figure 2C,D). This pivotal finding was further corroborated by immunofluorescence staining, which vividly described the cytoplasmic presence of Eef1a1 in both proliferating myoblasts and differentiated myotubes (Figure 2E). These collective outcomes suggested that Eef1a1 predominantly operates in post-transcriptional modifications within the cytoplasm. RT-qPCR analysis unveiled that Eef1a1 was notably enriched in slow-twitch soleus (SOL) muscle compared to fast-twitch tibialis anterior (TA) muscle, suggesting that Eef1a1 may influence muscle-fiber-type transition, but this needs to be further investigated (Figure 2F).

Building upon these compelling findings, we proceeded to establish a mouse model of muscle injury by administering cardiotoxin (CTX) injections into the tibialis anterior muscle, aiming to unravel the role of Eef1a1 in the intricate process of skeletal muscle regeneration (Figure S2A). A histological examination using hematoxylin and eosin (H&E) staining of the TA muscle unveiled a series of dynamic changes post-CTX injection. Notably, one day after the CTX injection, a significant dissolution of myofibers accompanied was observed. Subsequently, a rejuvenating response was evident with the emergence of new muscle fibers by day 7 post-injury. As the regeneration process advanced, the muscle fibers exhibited substantial repair by day 14, albeit with the persistence of a certain number of central nucleus muscle fibers (Figure S2B). Our scrutiny of markers during the course of muscle regeneration yielded intriguing insights. The expression level of the myosatellite cell marker Pax7 exhibited a pattern of initial increase followed by subsequent decrease, reflecting the temporal dynamics of muscle regeneration. Meanwhile, the expression profiles of the myogenic differentiation markers, MyoD and MyoG, showcased heightened expression during the later stages of skeletal muscle regeneration (Figure S2C). Remarkably, the expression pattern of Eef1a1 during muscle regeneration echoed a distinct trajectory. Rapid upregulation in the early stages was succeeded by a gradual decline as the regeneration process unfolded (Figure 2G). This trend suggested that Eef1a1 may hold a pivotal role as a potential regulator in the orchestration of skeletal muscle regeneration and myogenesis.

### 2.3. Eef1a1 Activates the Proliferation of Myoblasts

**Figure 3:**
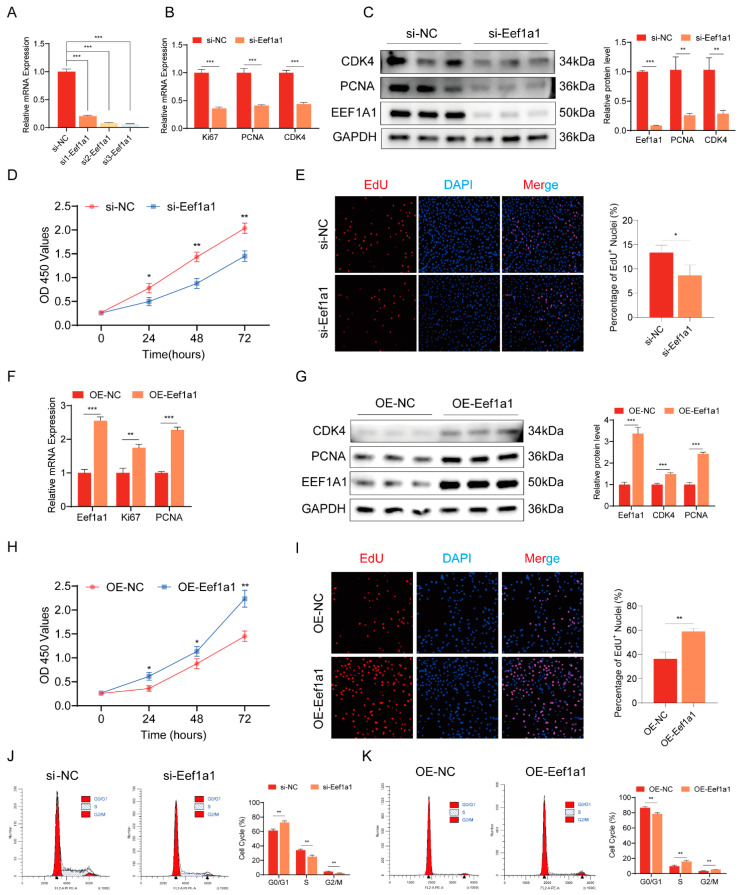
Eef1a1 promotes myogenic proliferation in C2C12 myoblasts. (**A**) Detection of interference efficiency in three small interfering Eef1a1(si-Eef1a1). (**B**,**C**) The mRNA and protein expression levels of myoblast proliferation marker genes from si-Eef1a1-transfected C2C12 myoblasts. (**D**) Cells were counted by CCK-8 after knockdown of Eef1a1. (**E**) EdU detection was performed after knockdown of Eef1a1. Cells in the process of DNA replication were labeled with EdU (red), whereas the cell nuclei were stained with DAPI (blue). The percentage of EdU+/nuclei (%) indicates the proportion of cells within the overall population that are currently undergoing DNA synthesis. Magnification 10×, scale bar 200 µm. (**F**,**G**) The mRNA and protein expression levels of myoblast proliferation marker genes from Eef1a1 overexpression (OE-Eef1a1) transfected C2C12 myoblasts. (**H**) Cells were counted by CCK-8 after overexpression of Eef1a1. (**I**) EdU detection was performed after overexpression of Eef1a1. Cells in the process of DNA replication were labeled with EdU (red), whereas the cell nuclei were stained with DAPI (blue). Magnification 10×, scale bar 200 µm. (**J**,**K**) Cell cycle distribution was detected by Flow cytometry. The RT-qPCR results were normalized against GAPDH expression. Error bars represent standard deviation and were derived from three independent experiments. * *p* < 0.05; ** *p* < 0.01; *** *p* < 0.001. EdU-positive cells were quantitated with ImageJ software (v1.46r).

To elucidate the functions of Eef1a1, we conducted interference and overexpression experiments to evaluate its role in myoblast proliferation. Three siRNAs targeting Eef1a1 were designed and transfected into C2C12 myoblasts, followed by RT-qPCR analysis. All three siRNAs showed high interference efficiency, among them, si3 was selected due to its highest knockdown efficiency (Figure 3A). The mRNA expression levels of the proliferation marker genes Ki67, PCNA, and CDK4 were evaluated using RT-qPCR in C2C12 myoblasts transfected with si3. The results demonstrated a significant decrease in the expression of Ki67, PCNA, and CDK4 following the knockdown of Eef1a1 (Figure 3B). Moreover, Western blot analysis confirmed that the protein levels of PCNA and CDK4 showed similar trends to the changes observed in mRNA expression (Figure 3C). To investigate the impact of Eef1a1 on the cell cycle of C2C12 cells, flow cytometry analysis was performed. The results revealed an increase in the proportion of G1-phase cells and a decrease in the proportion of S-phase cells following Eef1a1 knockdown (Figure 3J). Additionally, CCK-8 and EdU proliferation assays were conducted to assess the proliferation index of C2C12 myoblasts. The results indicated that Eef1a1 interference inhibited the proliferation of C2C12 myoblasts, as evidenced by the reduced proliferation index observed in both the CCK-8 assay (Figure 3D) and the proportion of EdU-positive cells (Figure 3E).

Additionally, we conducted overexpression experiments by transfecting Eef1a1 overexpression plasmids into C2C12 myoblasts. The successful overexpression of Eef1a1 was confirmed through RT-qPCR and Western blot analyses, which also revealed an increase in the expression of the proliferation marker genes Ki67 and PCNA during myoblast proliferation (Figure 3F,G). Cell cycle analysis following Eef1a1 overexpression demonstrated a reduction in the proportion of cells in the G1-phase and an increase in the proportion of cells in the S-phase (Figure 3K). Furthermore, EdU and CCK-8 proliferation assays provided additional support for the promotion of myoblast proliferation upon Eef1a1 overexpression. The percentage of EdU-positive cells increased (Figure 3I), and cell proliferation was enhanced (Figure 3H). Collectively, these findings provided evidence that Eef1a1 actively promotes the proliferation of C2C12 myoblasts.

### 2.4. Confirming the Function of Eef1a1 in Myoblast Proliferation through CRISPRko and CRISPRi

**Figure 4:**
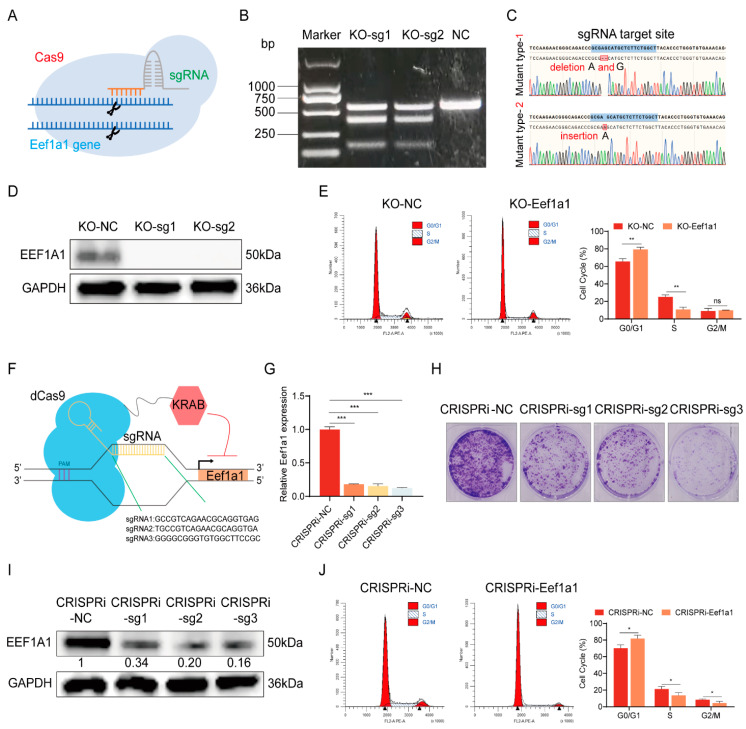
Functional exploration of cell proliferation regulation via CRISPR/Cas9- and CRISPRi-mediated Eef1a1 modulation. (**A**) Schematic diagram illustrating the CRISPR/Cas9-mediated Eef1a1 gene knockout process. (**B**) Diagram illustrating the T7E1 enzyme cleavage assay for validating the editing efficiency of the two sgRNAs. (**C**) Sanger sequencing chromatograms near the sgRNA target sites in the two Eef1a1 knockout cell lines. (**D**) Western blot results demonstrating successful Eef1a1 knockout in both cell lines. (**E**) Flow cytometry analysis of cell cycle distribution in Eef1a1 knockout cell lines. (**F**) Schematic diagram illustrating the use of CRISPRi technology to knock down Eef1a1 gene expression. (**G**) RT-qPCR and (**I**) Western blot analysis depicting the efficiency of Eef1a1 inhibition in CRISPRi cell lines, with relative signal intensities indicated below each panel. (**H**) Colony formation assay using crystal violet staining to validate the abundance of cell populations. (**J**) Flow cytometry sorting results illustrating the cell cycle distribution of cell lines with CRISPRi-mediated Eef1a1 knockdown. The RT-qPCR results were normalized against GAPDH expression. Error bars represent standard deviation and were derived from three independent experiments. * *p* < 0.05; ** *p* < 0.01, *** *p* < 0.001; ns, not significant.

To delve into the pivotal role of Eef1a1 in driving C2C12 myoblast proliferation, we harnessed the potential of the CRISPR/Cas9-based strategy, culminating in the generation of Eef1a1-KO C2C12 myoblasts (Figure 4A). Our high-throughput library screening proved fruitful as we successfully pinpointed two specific sgRNAs targeting Eef1a1. These sgRNAs were introduced into the C2C12-Cas9 cell line through transfection of the corresponding plasmid, and the ensuing T7E1 assay substantiated efficient gene editing efficacy for both sgRNAs (Figure 4B). The definitive establishment of two Eef1a1 knockout cell lines was confirmed through Sanger sequencing, where one exhibited deletions of bases A and G, while the other harbored an inserted base A (Figure 4C). Evidently, both indels introduced frameshift mutations culminating in the production of nonfunctional proteins. Immunoblotting analysis provided tangible evidence of the absence of the Eef1a1 protein in cells derived from both knockout lines (Figure 4D). Subsequently, the assessment of growth cycle dynamics in the KO-NC and KO-Eef1a1 cell lines, accomplished through flow cytometry, illuminated a pronounced increase in the proportion of cells in the G0/G1 phase coupled with a corresponding decline in the S phase upon Eef1a1 knockout (Figure 4E). These findings collectively underscored the role of Eef1a1 in driving C2C12 myoblast proliferation.

The CRISPR interference (CRISPRi) functional genomics platform, hinging on nuclease-inactive Cas9 (dCas9) fused with effector domains like KRAB, was harnessed to epigenetically repress gene expression. The elucidation of CRISPRi’s modus operandi was encapsulated in the schematic representation below (Figure 4F). In the context of our study, a trifecta of specific single guide RNAs (sgRNAs) were designed and implemented to generate the lentivirus-infected cell line, thereby achieving the targeted knockdown of the Eef1a1 gene. The validation through RT-qPCR (Figure 4G) and Western blot analyses (Figure 4I) offered unequivocal confirmation of successful Eef1a1 suppression. Insights garnered from colony formation assays provided clear-cut evidence, underscoring the significant dampening effect of Eef1a1 knockdown on the proliferation prowess of C2C12 (Figure 4H). Further delving into cell cycle dynamics through flow sorting, we observed that CRISPRi-mediated Eef1a1 knockdown mirrored the outcomes observed in the KO-Eef1a1 cell line. Flow sorting data showcased an analogous trend where the inhibition of Eef1a1 expression via CRISPRi engendered an augmented G0/G1-phase cell population, while concurrently witnessing a corresponding ebb in the S-phase pattern congruent with the findings from the KO-Eef1a1 cell line (Figure 4J). Collectively, these results coalesced to establish an important role of Eef1a1 in propelling myoblast proliferation.

### 2.5. Eef1a1 Inhibits Myoblast Differentiation and Fusion

**Figure 5:**
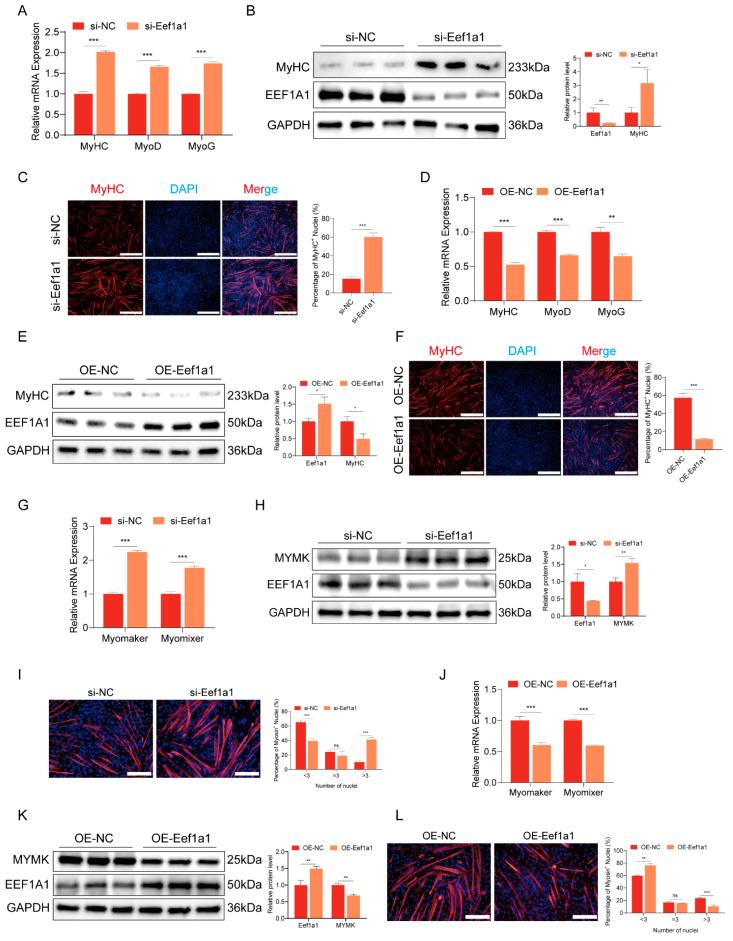
Eef1a1 inhibits myogenic differentiation and fusion in C2C12 myoblasts. (**A**,**B**) mRNA and protein expression levels of myoblast differentiation marker genes in C2C12 myoblasts transfected with Eef1a1 siRNA. (**C**) Immunofluorescence staining of C2C12 myoblasts differentiated for 3 days. Myotubes were labeled with MyHC (red) and cell nuclei were counterstained with DAPI (blue). Magnification 10×, scale bar 500 µm. (**D**,**E**) mRNA and protein expression levels of myoblast differentiation marker genes in C2C12 myoblasts transfected with OE-Eef1a1. (**F**) Immunofluorescence staining of C2C12 myoblasts differentiated for 3 days. Myotubes were labeled with MyHC (red) and cell nuclei were counterstained with DAPI (blue). Magnification 10×, scale bar 500 µm. (**G**,**H**) mRNA and protein expression levels of myoblast fusion marker genes in C2C12 myoblasts transfected with si-Eef1a1. (**I**) Immunofluorescence staining of C2C12 myoblasts differentiated for 5 days. Myotubes were labeled with MyHC (red) and cell nuclei were counterstained with DAPI (blue). Magnification 20×, scale bar 200 µm. (**J**,**K**) mRNA and protein expression levels of myoblast fusion marker genes in C2C12 myoblasts transfected with OE-Eef1a1. (**L**) Immunofluorescence staining of C2C12 myoblasts differentiated for 5 days. Myotubes were labeled with MyHC (red) and cell nuclei were counterstained with DAPI (blue). Magnification 20×, scale bar 200 µm. The RT-qPCR and Western blot results were normalized against GAPDH expression. Error bars represent standard deviation and were derived from three independent experiments. * *p* < 0.05; ** *p* < 0.01; *** *p* < 0.001; ns, not significant. MyHC-positive area, differentiation index, and fusion index were quantitated with ImageJ software. All experiments were repeated at least three times.

To further investigate the potential roles of Eef1a1, we initiated myoblast differentiation in C2C12 upon reaching confluence. This was achieved by substituting the growth medium with the differentiation medium. Subsequently, we employed a comprehensive approach involving RT-qPCR, Western blot analysis, and immunofluorescence staining to evaluate the expression of key myogenic marker genes, specifically MyoD, MyoG, and MyHC. Remarkably, interference with Eef1a1 significantly enhanced the myogenic differentiation of C2C12 myoblasts. This enhancement was substantiated by noteworthy increases in both the mRNA and protein levels of the myogenic marker genes (Figure 5A,B). Furthermore, our immunofluorescence staining results unveiled that the interference with Eef1a1 led to an augmentation in myoblast differentiation, prominently increasing the total area occupied by myotubes (Figure 5C). Conversely, the overexpression of Eef1a1 resulted in the reduced mRNA and protein expression levels of these myogenesis marker genes (Figure 5D–F).

Next, we further analyzed the function of Eef1a1 in the regulation of myotube fusion. Eef1a1 interference resulted in the increased mRNA expression of the fusion marker genes Myomaker (MYMK) and Myomixer (MYMX), as well as the increased protein expression of the fusion marker gene MYMK (Figure 5G,H). Immunofluorescence staining also revealed an increased proportion of myotubes with three or more nuclei (Figure 5I). In contrast, Eef1a1 overexpression inhibited the expression of these fusion marker genes and increased the proportion of myotubes with fewer than three cell nuclei (Figure 5J–L). These findings provided valuable insights into the role of Eef1a1 in regulating myoblast differentiation and myotube fusion processes.

### 2.6. miR-133a-3p Regulates Myogenesis by Targeting Eef1a1

**Figure 6:**
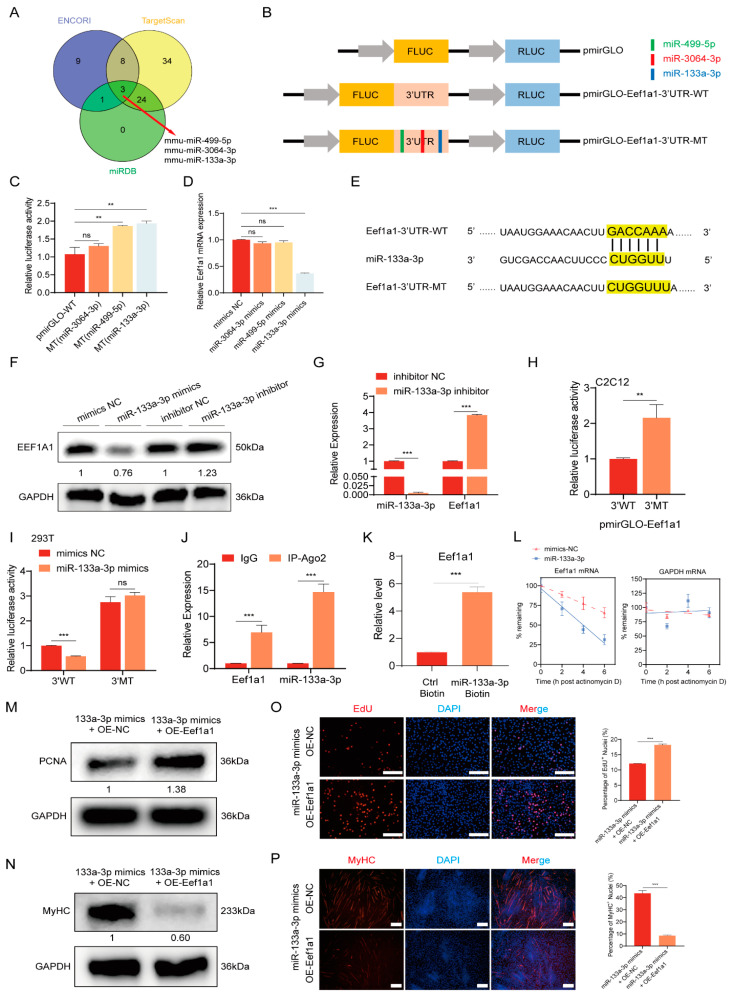
Eef1a1 is a direct target of miR-133a-3p. (**A**) Venn diagram showing the potential miRNA target predictions for Eef1a1 from different prediction tools. (**B**) Diagram illustrating the construction of dual-luciferase reporter vectors containing the wild-type or mutant Eef1a1 3′UTR sequences. (**C**) Relative luciferase activity assay in C2C12 myoblasts transfected with the Eef1a1 3′UTR wild-type or mutant dual-luciferase vector, measured 24 h later. (**D**) RT-qPCR analysis of Eef1a1 mRNA expression in C2C12 myoblasts after overexpression of miR-133a-3p and miR-499-5p. (**E**) The seed sequence of miR-133a-3p that targets Eef1a1, marked in yellow, along with the sequence of our constructed mutant vector. (**F**,**G**) mRNA and protein expression levels of Eef1a1 in C2C12 myoblasts transfected with miR-133a-3p mimics or inhibitors, with relative signal intensities indicated below each panel. (**H**) Dual luciferase assay results showing the effect of mutations in the miR-133a-3p target region on the 3′UTR of Eef1a1. (**I**) Relative luciferase activity assay in HEK293T cells co-transfected with miR-133a-3p mimics or mimics NC and the Eef1a1 3′UTR wild-type or mutant dual-luciferase vector, measured 24 h later. (**J**) RIP-qPCR experiment indicating the interaction between Eef1a1 and miR-133a-3p. (**K**) Transfections of biotinylated Ctrl and miR-133a-3p were conducted into C2C12 cells. After 24 h of transfection, RNA complexes were pulled down using streptavidin beads, and Eef1a1 in the pulled-down material was assessed by RT-qPCR analysis. (**L**) Cells were treated with actinomycin D 2 h, 4 h, and 6 h after transfection with miR-133a-3p mimics or mimics NC. Subsequently, the relative levels of Eef1a1 mRNA (left) and the stable control transcript GAPDH mRNA (right) were assessed by RT-qPCR analysis. The mRNA half-life (t1/2) was calculated as the time required to reach 50% of the initial mRNA abundance after the addition of actinomycin D. (**M**,**N**) Western blot analysis was conducted to assess the protein expression levels of PCNA and MyHC, with relative signal intensities indicated below each panel. (**O**) EdU detection was performed after co-transfected. Cells in the process of DNA replication were labeled with EdU (red), whereas the cell nuclei were stained with DAPI (blue). Magnification 10×, scale bar 200 µm. (**P**) Immunofluorescence staining in C2C12 myoblasts differentiated for 3 days. Myotubes were stained with MyHC (red) and cell nuclei were stained with DAPI (blue). Magnification 10×, scale bar 200 µm. The RT-qPCR results were normalized against GAPDH expression. Error bars represent standard deviation and were derived from three independent experiments. ** *p* < 0.01; *** *p* < 0.001; ns, not significant. EdU-positive cells were quantitated with ImageJ software. MyHC-positive area, and differentiation index were quantitated with ImageJ software.

MicroRNAs are small, non-coding RNAs that exert a significant influence on post-transcriptional gene regulation by binding to the 3′UTR of target genes. This interaction can result in either mRNA degradation or translation inhibition, thus modulating gene expression levels. To investigate the regulatory pattern of Eef1a1, we employed three different prediction tools, namely Targetscan (https://www.targetscan.org/mmu_80/, accessed on 6 March 2023), ENCORI (https://rnasysu.com/encori/, accessed on 6 March 2023), and miRDB (https://mirdb.org/mirdb/index.html, accessed on 6 March 2023), to identify potential miRNAs that could affect Eef1a1 expression. As a result, we found three miRNAs that bind to Eef1a1 (Figure 6A). To validate the targeting of these three potential miRNAs by Eef1a1, we cloned the Eef1a1 3‘UTR sequences downstream of the luciferase reporter gene (Figure 6B). The luciferase activity assays revealed that the overexpression of miR-133a-3p and miR-499-5p significantly repressed the luciferase activity of the wild-type (WT) Eef1a1 3′UTR reporter. However, these repressions were completely abolished when their corresponding binding sites were mutated (Figure 6C). Subsequently, we transfected mimics of miR-133a-3p and miR-499-5p into C2C12 myoblasts to verify their effects on Eef1a1 expression. Interestingly, only miR-133a-3p silenced the expression of Eef1a1, while miR-499-5p had no impact on Eef1a1 expression (Figure 6D). The seed sequence of miR-133a-3p perfectly matched the 3′UTR position of Eef1a1 mRNA and was highly conserved across species (Figure 6E). The transfection of miR-133a-3p mimics and inhibitors in C2C12 myoblasts was used to validate their effects on Eef1a1 at the RNA and protein levels. The results showed that the transfection of miR-133a-3p mimics inhibited Eef1a1 expression, while the transfection of miR-133a-3p inhibitors promoted Eef1a1 expression. It further illustrated that miR-133a-3p targets Eef1a1 (Figure 6F,G). The dual luciferase assay results indicated that when the miR-133a-3p target region in the 3‘UTR of Eef1a1 was mutated, the mutant group exhibited higher luciferase activity compared to the wild-type group (Figure 6H). To further confirm the interaction between miR-133a-3p and the Eef1a1 3′UTR, we transfected WT and mutant constructs into HEK293T cells along with miR-133a-3p mimic or a negative control (NC). The overexpression of miR-133a-3p significantly reduced the fluorescence activity in the WT construct, while the transfection of miR-133a-3p mimics in the mutant construct did not cause significant changes in fluorescence activity (Figure 6I). Moreover, the RIP-qPCR results for Ago2 protein demonstrated the significant enrichment of both Eef1a1 and miR-133a-3p compared to the IgG control group (Figure 6J). Biotinylated miR-133a-3p exhibited the ability to pull down approximately 5-fold more Eef1a1 compared to a biotinylated control miRNA (Figure 6K). These findings strongly suggest that Eef1a1 is a direct target gene of miR-133a-3p in muscle cells.

To delve deeper into how miR-133a-3p reduces Eef1a1 expression via the Eef1a1 3′UTR, we investigated whether miR-133a-3p regulates Eef1a1 mRNA stability. The method for calculating the half-life of Eef1a1 mRNA involves inhibiting de novo transcription using the RNA polymerase II inhibitor actinomycin D, then measuring the time required for Eef1a1 mRNA to decrease to half of its initial abundance [32]. Eef1a1 mRNA levels decreased more rapidly after miR-133a-3p overexpression (t1/2~4 h) compared to control groups (t1/2 > 6 h). Additionally, in the control experiment, GAPDH mRNA exhibited considerable stability (Figure 6L). To further investigate whether miR-133a-3p regulates the proliferation and differentiation of C2C12 myoblasts by targeting Eef1a1, we conducted a co-transfection assay using an Eef1a1 overexpression vector along with miR-133a-3p mimics. Western blot analysis revealed that the overexpression of miR-133a-3p could rescue the effects of Eef1a1 on the expression of myoblast proliferation and differentiation marker genes at the protein level (Figure 6M,N). The EdU assay showed that the overexpression of Eef1a1 increased the percentage of the EdU-positive cells, while this effect was reversed by miR-133a-3p mimics (Figure 6O). The immunofluorescence staining assay showed that Eef1a1 overexpression inhibited the myoblast differentiation capacity and significantly declined the total areas of myotubes, but co-transferring miR-133a-3p mimics would rescue this phenotype (Figure 6P).

## 3. Discussion

Muscle development and regeneration entail intricate and meticulously coordinated processes characterized by profound alterations in gene expression programs. Both RNA-binding proteins (RBPs) and non-coding RNAs (ncRNAs) play crucial roles in post-transcriptional regulation. Numerous studies underscore the pivotal involvement of non-coding RNAs, including long noncoding RNAs (lncRNAs) [33,34], microRNAs (miRNAs) [35,36,37], and circular RNAs (circRNAs) [38], in the intricate regulatory landscape governing muscle development. While the exploration of RNA-binding protein (RBP) regulation within the context of muscle development remains conspicuously limited.

Our investigation, using CRISPR library screening, identified Eef1a1 as an outstanding RNA-binding protein with a crucial role in myogenesis. Especially noteworthy was its impact on the proliferation rate of myoblasts. Through various methods such as siRNA, CRISPRko, and CRISPRi to individually knockdown the expression of Eef1a1, we observed a significant acceleration in the proliferation rate of myoblasts. In another study, researchers employed a Genome-wide CRISPR screen and successfully identified HNRNPL as a critical regulator of proliferation in LNCaP cells [39]. The screening approach used in their study was similar to ours. It was worth considering how to conduct high-throughput library screening related to muscle development using skeletal muscle stem cells in vivo. Some studies that utilize a CRISPR-Cas9 screen in vivo for discovering novel cancer targets were worth drawing inspiration from [25,27].

There are two isoforms of eEF1A, namely eEF1A1 and eEF1A2, both of which play similar roles in translation elongation—a critical step in protein synthesis. However, eEF1A1 and eEF1A2 exhibit differential expression patterns in tissues. While eEF1A1 is expressed ubiquitously, eEF1A2 shows preferential expression in skeletal muscle, heart, and brain tissues, indicating a distinct role for eEF1A2, particularly in muscle development [40,41]. Furthermore, in terminally differentiated myotubes, eEF1A1 has been demonstrated to promote apoptosis, while eEF1A2 played an anti-apoptotic role [42,43]. Our study showed that Eef1a1 promotes the proliferation of myoblasts, consistent with its regulatory role in promoting proliferation observed in cancer cells [44,45]. In our study, Eef1a1 plays a role in inhibiting myoblast differentiation. In a previous study, the eEF1A1 protein was found to be down-regulated during differentiation [43], implying that a reduction in eEF1A1 expression facilitates the progression of myoblast differentiation.

Additionally, we observed that Eef1a1 is primarily enriched in the soleus muscle, which predominantly consists of slow-twitch fibers. This suggested that Eef1a1 may play a potential role in oxidative metabolism, endurance, and the maintenance of muscle posture functions associated with slow-twitch fibers [46,47,48]. The expression of eEF1A2 in the fast and slow muscles of mdx (a DMD mouse model) mice showed no significant difference. This suggested that strategies focused on enhancing the expression and/or activity of eEF1A2 in muscles may offer therapeutic benefits for DMD (duchenne muscular dystrophy) patients [49]. Further investigation was needed to elucidate the role of Eef1a1 in the context of DMD. Eef1a1 expression was rapidly elevated in the short term after muscle injury. This indicated that Eef1a1 plays a significant role in the early days of muscle regeneration, suggesting its potential involvement in regulating the proliferation of skeletal muscle stem cells [50,51]. Furthermore, the amino acid sequence of Eef1a1 is conserved in mice, pigs, and humans, suggesting that this RBP may have potential applications in treating muscle diseases and improving meat production in animals. Therefore, our next step will involve further validating whether the function and mechanism of Eef1a1 is conserved in pig and human cells.

The mechanism of Eef1a1 regulating skeletal muscle development-related pathways may be related to the efficiency of the differential translation of some mRNA genes by Eef1a1. Consequently, within this mechanism, Eef1a1 may promote the translation of mRNA related to proliferation genes, or conversely, inhibit the translation of differentiation genes. MiRNAs, known for their potent regulatory roles, act by targeting the 3‘UTR of specific mRNA molecules. We demonstrated that Eef1a1 was a target gene of miR-133a-3p by dual luciferase, RIP assays, and miRNA pull-down assays. This muscle-enriched miRNA has previously been implicated in controlling the proliferation and differentiation of myogenic cells. Notably, our findings revealed a dynamic interplay between Eef1a1 and miR-133a-3p.

In conclusion, we identified the RNA-binding protein Eef1a1, which regulates myogenesis, through CRISPR library screening. Eef1a1 promotes myoblast proliferation and inhibits differentiation. Our results suggested that the miR-133a-3p/Eef1a1 pathway could be a novel mechanism for studying skeletal muscle growth and development. Our future investigation will focus on the role of Eef1a1 in regulating the mRNA translation of downstream genes. By elucidating these interactions, we aim to uncover the intricate network of genes modulated by Eef1a1 to orchestrate myogenic processes.

## 4. Materials and Methods

### 4.1. Construction of an RBP sgRNA Library Plasmid

The RBP sgRNA oligo pool was synthesized using CustomArray 12 K arrays (GenScript, Nanjing, China), followed by PCR amplification using KAPA HiFi HotStart ReadyMix (Roche, Basel, Switzerland) to generate sub-pools for In-Fusion Cloning (Vazyme, Nanjing, China). PCR reactions were conducted in a Veriti™ 96-Well Thermal Cycler (ABI, Waltham, MA, USA) for 20 cycles. Purification of PCR products was carried out using a MinElute PCR Purification Kit (QIAGEN, Dusseldorf, Germany) followed by ligation into linearized lenti-sgRNA-EGFP (addgene, #67975), lenti-sgRNA-puro (addgene, #104994), and lenti-sgRNA-BSD (addgene, #52962) vectors via In-Fusion Cloning. Ligation mixtures were transformed into Endur^TM^ Electrocompetent Cells (Lucigen, Middleton, WI, USA) through parallel transformations, ensuring a colony count of at least 300 times the total number of sgRNAs in the library for comprehensive coverage. Subsequently, sgRNA library plasmids were extracted using the Endo-Free Plasmid Maxi Kit (OMEGA, Stratford, WI, USA). Amplification of library plasmids was performed using KAPA HiFi HotStart ReadyMix (Roche, Basel, Switzerland) with 22 reaction cycles. PCR products were purified using a Gel and PCR Clean Up Kit (OMEGA, Stratford, WI, USA) and then subjected to high-throughput sequencing to evaluate sgRNA coverage within the library plasmids.

### 4.2. Construction of RBP Knockout Cell Libraries and Screening

Initially, the plasmids encoding Cas9 along with pMD2.G and psPAX2 lentiviral plasmids (VectorBuilder, Guangzhou, China) were transfected into 293T cells. After a 60 h incubation period, the supernatant containing the generated lentivirus was harvested and utilized to infect the target cells, C2C12. Subsequent to drug screening for positive cells, monoclonal cell sorting and expansion procedures were carried out, culminating in obtaining a C2C12 monoclonal cell line expressing Cas9. C2C12-Cas9 cells were seeded into T75 flasks and then infected with the library lentiviruses at a multiplicity of infection (MOI) of 0.3. Three days post-infection, GFP-positive cells were sorted using Fluorescence-Activated Cell Sorting (FACS), while puromycin-resistant cells were selected with 1.5 μg/mL puromycin. Subsequently, the sorted (1B) and selected cells (2B) were reseeded into T75 flasks. The cells would then be divided into two groups, one was collected after 1 day of proliferation and the other was collected when the cells were left for continuous proliferation in the growth medium for 5 days.

### 4.3. Illumina Sequencing of sgRNAs

The genomic DNA of each sample was extracted using a Blood and Cell Culture DNA Midi Kit (QIAGEN, Dusseldorf, Germany). The sgRNA region was amplified by PCR using Q5^®^ Hot Start High-Fidelity DNA Polymerase (NEB, Milwaukee, WI, USA) in a reaction volume of 50 μL. PCR products were mixed and purified with a MinElute PCR purifcation Kit (QIAGEN, Dusseldorf, Germany), followed by Illumina Novaseq6000 PE150.

### 4.4. Differential sgRNA Enrichment Analysis

After sequencing, reads for each sgRNA were counted and the percentage of each sgRNA relative to the total number of sgRNA reads was calculated. The changes in the percentage of sgRNAs were then compared to identify enrichment in up-regulation or down-regulation. sgRNAs with a false discovery rate (FDR) ≤ 0.05 and |log2FoldChange| ≥ 1 were considered as differentially expressed sgRNAs. Up-regulated sgRNAs indicate a decrease in the expression level of the corresponding gene.

### 4.5. Cell Culture and Transfection

The C2C12 myoblasts were sourced from the American Type Culture Collection (ATCC, Manassas, VA, USA). The growth medium (GM) consisted of Dulbecco’s Modified Eagle’s Medium basic (DMEM, Gibco, Pleasanton, CA, USA) supplemented with 10% fetal bovine serum (FBS, ExCell Bio, Shanghai, China) and 1% penicillin–streptomycin (PS, Thermo Scientific, Waltham, MA, USA). For differentiation, DMEM medium was used, containing 2% horse serum (Biological Industries, Kibbutz Beit Haemek, Israel) and 1% PS (DM). Cells were maintained in a 37 °C cell incubator under a controlled atmosphere of 5% carbon dioxide. Transfections were performed using the jetPRIME (Polyplus, Illkirch, France) transfection reagent following the manufacturer’s instructions.

### 4.6. Plasmid Construction, siRNA, and microRNA Synthesis

RiboBio (Guangzhou, China) was responsible for the design and synthesis of small interfering RNAs (siRNAs) targeting Eef1a1, as well as non-specific siRNA negative controls, to achieve efficient knockdown of Eef1a1. Additionally, RiboBio synthesized miR-133a-3p mimic, mimic NC, miR-133a-3p inhibitor, and inhibitor NC for functional studies. To establish the Eef1a1 overexpression system, the full-length coding sequence (CDS) of Eef1a1 was amplified from mouse anterior tibialis muscle cDNA using PCR and subsequently cloned into the pcDNA3.1 expression plasmid vector using the NheI and XhoI restriction sites. Furthermore, experimental assays involving the wild-type and mutant pmirGLO Dual-Luciferase vectors were performed, and these vectors were provided by Wuhan Genecreate. Paired oligonucleotides of sgRNA were annealed and cloned into the lenti-sgRNA vector at the BbsI site. All constructed plasmids were subjected to Sanger sequencing for sequence validation. The primers for all plasmids were provided in Table S5.

### 4.7. RNA Extraction and RT-qPCR

Total RNA from cells and tissues was extracted using the Trizol method (Invitrogen, Shanghai, China). The purity and concentration of RNA were determined using a NanoDrop 2000 spectrophotometer (Thermo Fisher Scientific, Waltham, MA, USA). Generally, RNA samples with an OD260/OD280 ratio between 1.8 and 2.0 are considered to be of good quality for subsequent experiments. The total RNA was reverse transcribed into cDNA according to the instructions of the reverse transcription kit (Vazyme, Nanjing, China). Quantitative real-time PCR (RT-qPCR) was performed on an ABI Step One Plus Real-Time PCR system (Applied Biosystems, Norwalk, CT, USA) using Fast ChamQ Universal SYBR qPCR Master Mix (Vazyme, Nanjing, China), following the manufacturer’s instructions. The 2^−ΔΔCt^ approach was utilized to assess the relative expression levels of both miRNA and mRNA. U6 and GAPDH were used as endogenous controls to normalize the expression of miRNA and mRNA, respectively. The sequence information of primers used for reverse transcription and quantification was listed in Table S6.

### 4.8. Western Blot Analysis

Cell samples were collected and total proteins were extracted from the cells using protein lysis buffer (Thermo Scientific, USA). The protein concentration was determined using the BCA assay (Beyotime, Shanghai, China). Subsequently, protein gel electrophoresis and protein immunoblotting were performed using SDS-PAGE gels (EpiZyme, Shanghai, China) and 0.45 µm NC membranes (Merck, Rahway, NY, USA). The NC membranes were then blocked with 5% skim milk at room temperature for 2 h. After washing the membranes with PBS, they were incubated with primary antibodies for 3 h at room temperature (or overnight at 4 °C), followed by incubation with secondary antibodies for 1 h. This study employed the following antibodies: GAPDH (1:1000, ab9482, Abcam, Cambridge, UK), EEF1A1 (1:2000, 11402-1-AP, Proteintech Group, Wuhan, China), PCNA (1:1000, ab18197, Abcam, UK), CDK4 (1:1000, 11026-1-AP, Proteintech Group, China), MyHC (1:1000, MF20, DSHB, Iowa City, IA, USA), MYMK (1:500, A18158, ABclonal, Wuhan, China), Goat Anti-Mouse (ZB-2305, 1:1000, ZSGB-BIO, Beijing, China), and Goat Anti-Rabbit (ZB-2301, 1:1000, ZSGB-BIO, Beijing, China).

### 4.9. Cell Proliferation Assay (CCK-8)

Equal amounts of cells were seeded into four 96-well plates, respectively. Transfection was performed after starvation treatment, once the cell density reached 50%. After 24 h of transfection, 10 µL of CCK-8 reagent (C0038, Beyotime, Shanghai, China) was added according to the instructions. Cells were harvested at 0 h, 24 h, 48 h, and 72 h, and the absorbance at 450 nm was measured using a microplate reader.

### 4.10. Cell Proliferation EdU Staining Assay

The cells were seeded in a 12-well plate and cell transfection was conducted when the cell density reached around 50%. After 48 h of transfection, 2× EdU working solution was added for EdU staining (Beyotime, Shanghai, China). Following a 2 h incubation at 37 °C in a cell culture incubator, cells were fixed with 4% paraformaldehyde (Beyotime, Shanghai, China) at room temperature for 15 min and permeabilized with 0.5% Triton X-100 for 10 min. Subsequently, the Click Additive Solution was added and cells were incubated in the dark for 30 min. Finally, cells were incubated with 4′,6-diamidino-2-phenylindole (DAPI) (Sigma, Livonia, MI, USA) for 10 min and images were observed and captured using a Nikon ECLIPSE Ti microscope (Japanese).

### 4.11. Colony Formation Assay

Both the wild-type and Eef1a1-CRISPRi silenced cell lines were prepared for the assay by trypsinizing and counting using a hemocytometer. Subsequently, 200 cells from each cell line were seeded into individual wells of 6-well tissue culture plates using proliferation medium. The plates were incubated under standard cell culture conditions for 5 days, and the medium was refreshed every 2–3 days throughout the incubation period. Following the 5-day incubation, Crystal Violet staining (Beyotime, Shanghai, China) was performed to visualize and assess changes in cell colony numbers within each well.

### 4.12. Flow Cytometric Analysis of the Cell Cycle

The cultured myobalsts in growth media were collected 48 h after transfection. The cells were then fixed overnight at 4 °C in 70% ethanol, following the protocol of the Cell Cycle and Apoptosis Assay Kit (Beyotime, Shanghai, China). Subsequently, the cell precipitate was treated with propidium iodide (PI) solution and incubated at 37 °C for 30 min in the dark. Finally, the samples were analyzed using Beckman Coulter flow cytometry (Indianapolis, IN, USA).

### 4.13. RNA Stability Analyses

Actinomycin D (ActD, APExBIO, Houston, TX, USA) was used to analyze the effect of miR-133a-3p on Eef1a1 mRNA stability. Twenty-four hours after transfecting mimics NC or miR-133a-3p mimics, cells were then treated with actinomycin D for 0 h, 2 h, 4 h, and 6 h. The relative levels of Eef1a1 mRNA and GAPDH mRNA (a control stable transcript) were evaluated using RT-qPCR analysis. Additionally, mRNA half-lives (t1/2) were determined as the times required to reach 50% of the initial mRNA abundance at time 0, before the addition of actinomycin D, also analyzed by RT-qPCR.

### 4.14. Immunofluorescence Assay

C2C12 cells were cultured and differentiated as required, followed by fixation in 4% paraformaldehyde for 30 min. Subsequently, the cells were permeabilized using 0.5% Triton X-100 for 20 min and then blocked with 5% bovine serum albumin (BSA, Solarbio, Beijing, China) for 1 h. The primary antibody (1:500, MF20, DSHB, USA) was incubated at room temperature for 2 h. Next, the cells were exposed to the secondary antibody (1:500, GB121152-100, Servicebio, Wuhan, China) at room temperature, protected from light, for 1 h. Nuclei were labeled with DAPI for 10 min and the images were observed and captured using a Nikon ECLIPSE Ti microscope.

### 4.15. Dual-Luciferase Reporter Assay

The wild-type pmirGLO vector containing the Eef1a1 3‘UTR sequence, along with mutant pmirGLO vectors containing potential target site mutations for miR-133a-3p, miR-499-5p, and miR-3064-3p, were individually transfected into separate wells of a 6-well tissue culture plate using a plasmid quantity of 2 μg per well. Additionally, to further confirm the interaction between Eef1a1 and miR-133a-3p, the wild-type pmirGLO Dual-Luciferase miRNA target expression vector of Eef1a1 was co-transfected into HEK293T cells with miR-133a-3p mimics. After a 36 h transfection period, cells were treated according to the Dual-Luciferase^®^ Reporter Assay System (Promega, Madison, WI, USA). The Firefly luciferase/Renillaluciferase ratio was obtained by a GloMax 20/20 Luminometer (Promega, Madison, WI, USA).

### 4.16. Muscle Injury and Regeneration

A regeneration model was constructed by injecting 50 μL of 20 μM cardiotoxin (CTX) (217503, Sigma-Aldrich, St. Louis, MO, USA) into the tibialis anterior (TA) muscle of 7-week-old SPF-grade C57BL/6 male mice, which resulted in the injury of mouse skeletal muscle. Muscle samples were dissected and collected at 0 d, 1 d, 3 d, 5 d, 7 d, and 14 d after injury for RNA extraction and tissue sectioning, respectively.

### 4.17. Nuclear and Cytoplasmic RNA and Protein Fractionation

Proliferation phase cells were harvested at a density of 80%. Differentiation stage cells were harvested at 3 days of induced differentiation. Nuclear and cytoplasmic RNA fractionation was performed using the Cytoplasmic and Nuclear RNA Purification Kit (Norgen Biotek, Wuhan, China). Nuclear and cytoplasmic protein fractionation were performed using the ExKine™ Nuclei Extraction Kit (Abbkine, Wuhan, China). The specific operation steps were conducted according to the reference instructions. Quantification of cytoplasmic and nuclear RNA distribution was conducted by RT-qPCR analysis. Quantification of cytoplasmic and nuclear protein was performed by Western blot.

### 4.18. RNA Immunoprecipitation (RIP) Assay

C2C12 myoblasts were seeded into a T75 cell culture flask and transfected with miR-133a-3p mimics. Cells were harvested 48 h post-transfection. The RIP experiment was conducted following the guidelines of the Magna RIP™RNA-Binding Protein Immunoprecipitation Kit (Millipore, Burlington, MA, USA), with miRNA being pulled down using an anti-Ago2 antibody (ab186733, Abcam, Cambridge, UK). Enrichment multiples of miR-133a-3p and Eef1a1 were detected by RT-qPCR.

### 4.19. Biotin-Labeled Pull-Down Assays

Biotinylated miR-133a-3p was transfected into C2C12 cells. After the cells were collected, they were incubated with lysis buffer for 20 min. The cytoplasmic lysates were then incubated together with streptavidin magnetic beads (M-280, Dynabeads, Life Technologies, Waltham, MA, USA) for 4 h. Following the washing of pull-down samples with lysis buffer, RNA isolation from the pull-down was performed using the Direct-zol™ RNA MiniPrep Kit (Zymo Research, Orange, CA, USA), selected for its capability in RNA separation.

### 4.20. Statistical Analysis

All experiments in this study were repeated at least three times and statistical analyses of the data were performed using GraphPad Prism 5.0 software. The statistical methods included *t*-tests and multifactorial analysis of variance. Results are presented as mean ± SD, with differences considered statistically significant at *p* < 0.05. Asterisks denote significance levels as follows: * for *p* < 0.05, ** for *p* < 0.01, *** for *p* < 0.001, and “ns” indicates non-significant differences (*p* ≥ 0.05).

## Data Availability

The data used to support the findings of this study are included within the article. Raw data are available on request from the corresponding author.

## Supplementary Materials

The following supporting information can be downloaded at: https://www.mdpi.com/article/10.3390/ijms25094816/s1.

## References

[1] Shen L., Du J., Xia Y., Tan Z., Fu Y., Yang Q., Li X., Tang G., Jiang Y., Wang J. (2016). Genome-wide landscape of DNA methylomes and their relationship with mRNA and miRNA transcriptomes in oxidative and glycolytic skeletal muscles. Sci. Rep..

[2] Scott L.J., Erdos M.R., Huyghe J.R., Welch R.P., Beck A.T., Wolford B.N., Chines P.S., Didion J.P., Narisu N., Stringham H.M. (2016). The genetic regulatory signature of type 2 diabetes in human skeletal muscle. Nat. Commun..

[3] Sartori R., Romanello V., Sandri M. (2021). Mechanisms of muscle atrophy and hypertrophy: Implications in health and disease. Nat. Commun..

[4] Sousa-Victor P., García-Prat L., Muñoz-Cánoves P. (2021). Control of satellite cell function in muscle regeneration and its disruption in ageing. Nat. Rev. Mol. Cell Biol..

[5] Yamamoto M., Legendre N.P., Biswas A.A., Lawton A., Yamamoto S., Tajbakhsh S., Kardon G., Goldhamer D.J. (2018). Loss of MyoD and Myf5 in Skeletal Muscle Stem Cells Results in Altered Myogenic Programming and Failed Regeneration. Stem Cell Rep..

[6] Hernandez-Hernandez M., Garcia-Gonzalez E.G., Brun C.E., Rudnicki M.A. (2017). The myogenic regulatory factors, determinants of muscle development, cell identity and regeneration. Semin. Cell Dev. Biol..

[7] Chal J., Pourquie O. (2017). Making muscle: Skeletal myogenesis in vivo and in vitro. Development.

[8] Bentzinger C.F., Wang Y.X., Rudnicki M.A. (2012). Building Muscle: Molecular Regulation of Myogenesis. Cold Spring Harb. Perspect. Biol..

[9] Seale P., Sabourin L.A., Girgis-Gabardo A., Mansouri A., Gruss P., Rudnicki M.A. (2000). Pax7 Is Required for the Specification of Myogenic Satellite Cells. Cell.

[10] Buckingham M., Relaix F. (2015). PAX3 and PAX7 as upstream regulators of myogenesis. Semin. Cell Dev. Biol..

[11] Cooper R.N., Tajbakhsh S., Mouly V., Cossu G., Buckingham M., Butler-Browne G.S. (1999). In vivo satellite cell activation via Myf5 and MyoD in regenerating mouse skeletal muscle. J. Cell Sci..

[12] Li L., Olson E.N. (1992). Regulation of muscle cell growth and differentiation by the MyoD family of helix-loop-helix proteins. Adv. Cancer Res..

[13] Yue B., Yang H., Wang J., Ru W., Wu J., Huang Y., Lan X., Lei C., Chen H. (2020). Exosome biogenesis, secretion and function of exosomal miRNAs in skeletal muscle myogenesis. Cell Prolif..

[14] Li Y., Zhang Y., Hu Q., Egranov S.D., Xing Z., Zhang Z., Liang K., Ye Y., Pan Y., Chatterjee S.S. (2021). Functional significance of gain-of-function H19 lncRNA in skeletal muscle differentiation and anti-obesity effects. Genome Med..

[15] Yan J., Yang Y., Fan X., Liang G., Wang Z., Li J., Wang L., Chen Y., Adetula A.A., Tang Y. (2021). circRNAome profiling reveals circFgfr2 regulates myogenesis and muscle regeneration via a feedback loop. J. Cachex.-Sarcopenia Muscle.

[16] Yang W., Yang L., Wang J., Zhang Y., Li S., Yin Q., Suo J., Ma R., Ye Y., Cheng H. (2021). Msi2-mediated MiR7a-1 processing repression promotes myogenesis. J. Cachex.-Sarcopenia Muscle.

[17] Nguyen M.T., Lee W. (2022). MiR-320-3p Regulates the Proliferation and Differentiation of Myogenic Progenitor Cells by Modulating Actin Remodeling. Int. J. Mol. Sci..

[18] Yan S., Pei Y., Li J., Tang Z., Yang Y. (2023). Recent Progress on Circular RNAs in the Development of Skeletal Muscle and Adipose Tissues of Farm Animals. Biomolecules.

[19] Zhang M., Han Y., Liu J., Liu L., Zheng L., Chen Y., Xia R., Yao D., Cai X., Xu X. (2020). Rbm24 modulates adult skeletal muscle regeneration via regulation of alternative splicing. Theranostics.

[20] Apponi L.H., Corbett A.H., Pavlath G.K. (2011). RNA-binding proteins and gene regulation in myogenesis. Trends Pharmacol. Sci..

[21] Fujita R., Crist C. (2018). Translational Control of the Myogenic Program in Developing, Regenerating, and Diseased Skeletal Muscle. Curr. Top. Dev. Biol..

[22] Gebauer F., Schwarzl T., Valcárcel J., Hentze M.W. (2020). RNA-binding proteins in human genetic disease. Nat. Rev. Genet..

[23] Zhao C., Liu H., Xiao T., Wang Z., Nie X., Li X., Qian P., Qin L., Han X., Zhang J. (2020). CRISPR screening of porcine sgRNA library identifies host factors associated with Japanese encephalitis virus replication. Nat. Commun..

[24] Chen S., Sanjana N.E., Zheng K., Shalem O., Lee K., Shi X., Scott D.A., Song J., Pan J.Q., Weissleder R. (2015). Genome-wide CRISPR Screen in a Mouse Model of Tumor Growth and Metastasis. Cell.

[25] Bajaj J., Hamilton M., Shima Y., Chambers K., Spinler K., Van Nostrand E.L., Yee B.A., Blue S.M., Chen M., Rizzeri D. (2020). An in vivo genome-wide CRISPR screen identifies the RNA-binding protein Staufen2 as a key regulator of myeloid leukemia. Nat. Cancer.

[26] Jaitin D.A., Weiner A., Yofe I., Lara-Astiaso D., Keren-Shaul H., David E., Salame T.M., Tanay A., van Oudenaarden A., Amit I. (2016). Dissecting Immune Circuits by Linking CRISPR-Pooled Screens with Single-Cell RNA-Seq. Cell.

[27] Wang X., Tokheim C., Gu S.S., Wang B., Tang Q., Li Y., Traugh N., Zeng Z., Zhang Y., Li Z. (2021). In vivo CRISPR screens identify the E3 ligase Cop1 as a modulator of macrophage infiltration and cancer immunotherapy target. Cell.

[28] Zhou Q., Zhan H., Liao X., Fang L., Liu Y., Xie H., Yang K., Gao Q., Ding M., Cai Z. (2018). A revolutionary tool: CRISPR technology plays an important role in construction of intelligentized gene circuits. Cell Prolif..

[29] Konermann S., Brigham M.D., Trevino A.E., Joung J., Abudayyeh O.O., Barcena C., Hsu P.D., Habib N., Gootenberg J.S., Nishimasu H. (2014). Genome-scale transcriptional activation by an engineered CRISPR-Cas9 complex. Nature.

[30] Black J.B., McCutcheon S.R., Dube S., Barrera A., Klann T.S., Rice G.A., Adkar S.S., Soderling S.H., Reddy T.E., Gersbach C.A. (2020). Master Regulators and Cofactors of Human Neuronal Cell Fate Specification Identified by CRISPR Gene Activation Screens. Cell Rep..

[31] Przybyla L., Gilbert L.A. (2021). A new era in functional genomics screens. Nat. Rev. Genet..

[32] Yang J.H., Chang M.W., Tsitsipatis D., Yang X., Martindale J.L., Munk R., Cheng A., Izydore E., Pandey P.R., Piao Y. (2022). LncRNA OIP5-AS1-directed miR-7 degradation promotes MYMX production during human myogenesis. Nucleic Acids Res..

[33] Zhu M., Liu J., Xiao J., Yang L., Cai M., Shen H., Chen X., Ma Y., Hu S., Wang Z. (2017). Lnc-mg is a long non-coding RNA that promotes myogenesis. Nat. Commun..

[34] Wang S., Zuo H., Jin J., Lv W., Xu Z., Fan Y., Zhang J., Zuo B. (2019). Long noncoding RNA Neat1 modulates myogenesis by recruiting Ezh2. Cell Death Dis..

[35] Wang Y.-C., Yao X., Ma M., Zhang H., Wang H., Zhao L., Liu S., Sun C., Li P., Wu Y. (2021). miR-130b inhibits proliferation and promotes differentiation in myocytes via targeting Sp1. J. Mol. Cell Biol..

[36] Liu Y., Yao Y., Zhang Y., Yan C., Yang M., Wang Z., Li W., Li F., Wang W., Yang Y. (2023). MicroRNA-200c-5p Regulates Migration and Differentiation of Myoblasts via Targeting Adamts5 in Skeletal Muscle Regeneration and Myogenesis. Int. J. Mol. Sci..

[37] Zhang Y., Yao Y., Wang Z., Lu D., Zhang Y., Adetula A.A., Tang Z., Yi G., Liu Y., Chen Y. (2022). MiR-743a-5p regulates differentiation of myoblast by targeting Mob1b in skeletal muscle development and regeneration. Genes Dis..

[38] Yan J., Yang Y., Fan X., Tang Y., Tang Z. (2021). Sp1-Mediated circRNA circHipk2 Regulates Myogenesis by Targeting Ribosomal Protein Rpl7. Genes.

[39] Fei T., Chen Y., Xiao T., Li W., Cato L., Zhang P., Cotter M.B., Bowden M., Lis R.T., Zhao S.G. (2017). Genome-wide CRISPR screen identifies HNRNPL as a prostate cancer dependency regulating RNA splicing. Proc. Natl. Acad. Sci. USA.

[40] Knudsen S.M., Frydenberg J., Clark B.F.C., Leffers H. (1993). Tissue-dependent variation in the expression of elongation factor-1α isoforms: Isolation and characterisation of a cDNA encoding a novel variant of human elongation-factor 1α. JBIC J. Biol. Inorg. Chem..

[41] Lund A., Knudsen S., Vissing H., Clark B., Tommerup N. (1996). Assignment of Human Elongation Factor 1α Genes:EEF1AMaps to Chromosome 6q14 andEEF1A2to 20q13.3. Genomics.

[42] Lee S., Francoeur A., Liu S., Wang E. (1992). Tissue-specific expression in mammalian brain, heart, and muscle of S1, a member of the elongation factor-1 alpha gene family. J. Biol. Chem..

[43] Ruest L.-B., Marcotte R., Wang E. (2002). Peptide Elongation Factor eEF1A-2/S1 Expression in Cultured Differentiated Myotubes and Its Protective Effect against Caspase- 3-mediated Apoptosis. J. Biol. Chem..

[44] Liu X., Chen L., Ge J., Yan C., Huang Z., Hu J., Wen C., Li M., Huang D., Qiu Y. (2016). The Ubiquitin-like Protein FAT10 Stabilizes eEF1A1 Expression to Promote Tumor Proliferation in a Complex Manner. Cancer Res..

[45] Cui H., Li H., Wu H., Du F., Xie X., Zeng S., Zhang Z., Dong K., Shang L., Jing C. (2022). A novel 3’tRNA-derived fragment tRF-Val promotes proliferation and inhibits apoptosis by targeting EEF1A1 in gastric cancer. Cell Death Dis..

[46] Qin H., Ni H., Liu Y., Yuan Y., Xi T., Li X., Zheng L. (2020). RNA-binding proteins in tumor progression. J. Hematol. Oncol..

[47] Xu M., Chen X., Huang Z., Chen D., Chen H., Luo Y., Zheng P., He J., Yu J., Yu B. (2020). Procyanidin B2 Promotes Skeletal Slow-Twitch Myofiber Gene Expression through the AMPK Signaling Pathway in C2C12 Myotubes. J. Agric. Food Chem..

[48] Hayashi T., Fujita R., Okada R., Hamada M., Suzuki R., Fuseya S., Leckey J., Kanai M., Inoue Y., Sadaki S. (2023). Lunar gravity prevents skeletal muscle atrophy but not myofiber type shift in mice. Commun. Biol..

[49] Péladeau C., Adam N., Bronicki L.M., Coriati A., Thabet M., Al-Rewashdy H., Vanstone J., Mears A., Renaud J.-M., Holcik M. (2020). Identification of therapeutics that target eEF1A2 and upregulate utrophin A translation in dystrophic muscles. Nat. Commun..

[50] Wang C., Zhu Y., Wu D., Wang Z., Xu X., Shi Y., Yang G., Yu Y., Peng X. (2020). The role of PDIA3 in myogenesis during muscle regeneration. Exp. Mol. Med..

[51] Xie S.-J., Lei H., Yang B., Diao L.-T., Liao J.-Y., He J.-H., Tao S., Hu Y.-X., Hou Y.-R., Sun Y.-J. (2021). Dynamic m6A mRNA Methylation Reveals the Role of METTL3/14-m6A-MNK2-ERK Signaling Axis in Skeletal Muscle Differentiation and Regeneration. Front. Cell Dev. Biol..

