# Supplementary material for: CRISPR Screen Identifies the RNA-Binding Protein Eef1a1 as a Key Regulator of Myogenesis
Source: Int J Mol Sci. 2024 Apr 28;25(9):4816. doi: 10.3390/ijms25094816 (PMC11084334; doi:10.3390/ijms25094816)
Supplement: Supplementary file 1 [file ijms-25-04816-s001.zip › Supplementary Figure.pdf]

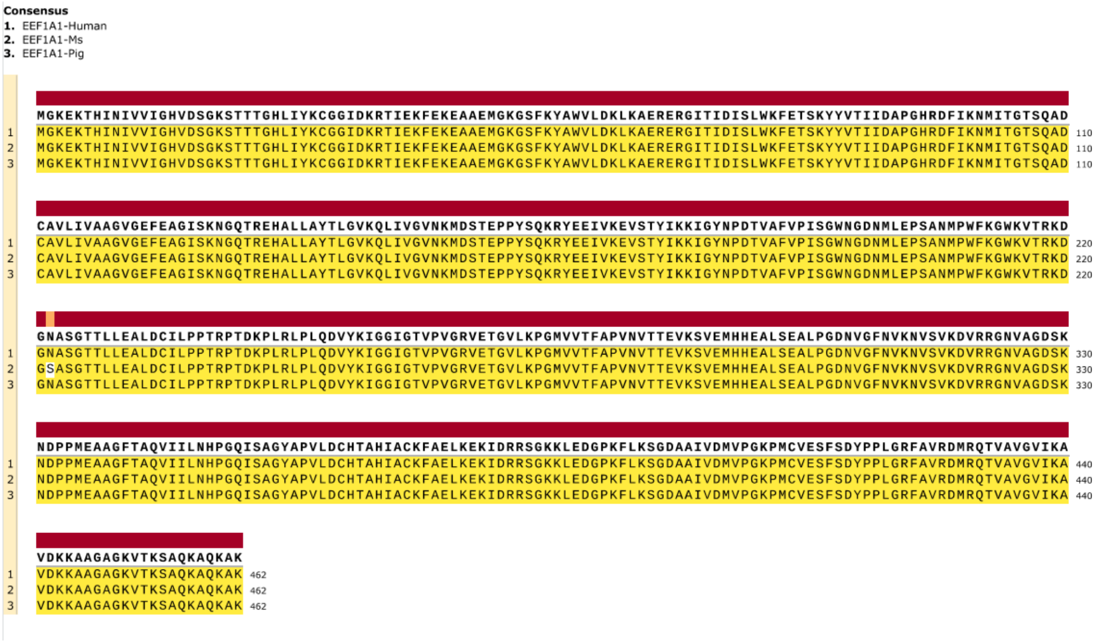

Figure S1. Amino acid sequence alignment of Eef1a1 across diverse species (human, mouse and pig), highlighting conserved regions.

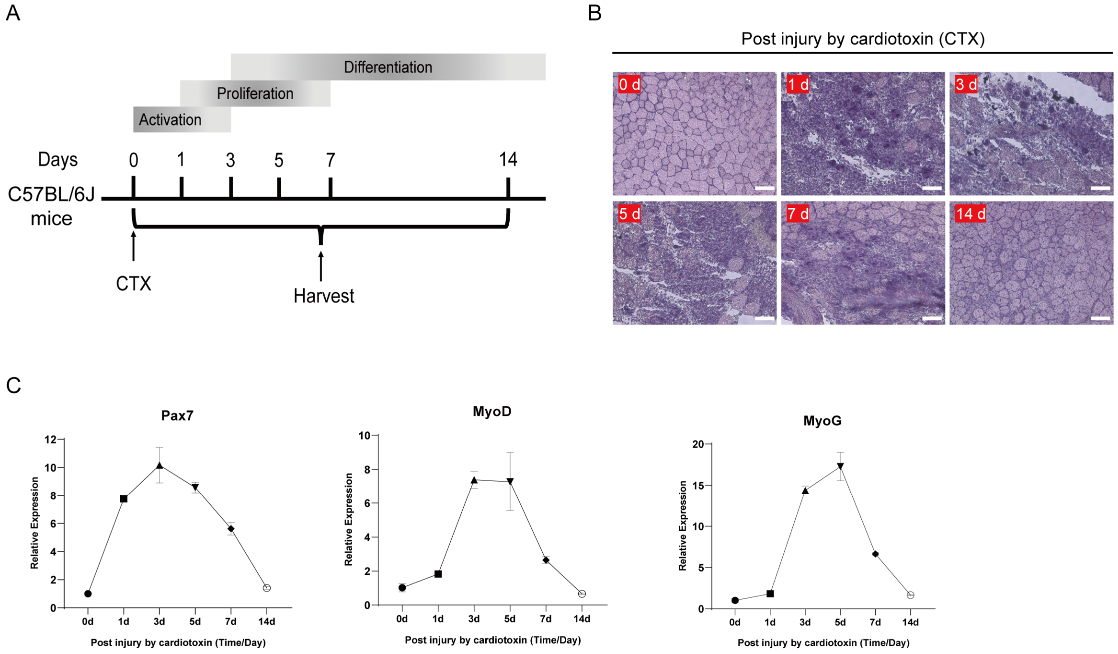

Figure S2. The process of muscle regeneration. (A) Diagram depicting the muscle injury model used for investigations. (B) Hematoxylin and eosin (HE) staining of mouse tibialis anterior (TA) muscle sections, revealing the morphological changes post-injury. Magnification 10×, scale bar 200 μm. (C) Expression patterns of marker gene in TA muscle of mice during the process of muscle regeneration. The RT-qPCR results were normalized to GAPDH. Error bars indicated standard deviation and were based on three independent experiments. *$P < 0.05$; **$P < 0.01$; ***$P < 0.001$ns, no significant. All experiments were repeated at least three times.
